# Case Report: Dupilumab in difficult-to-treat bullous pemphigoid at the University Hospital Schleswig-Holstein, Campus Kiel

**DOI:** 10.3389/fmed.2025.1697048

**Published:** 2026-01-23

**Authors:** Y. Mehrjerdian, R. Atallah, B. Wijaya, C. M. Hammers, S. Gerdes, G. Heine

**Affiliations:** 1 Department of Dermatology, Venereology and Allergy, University Hospital Schleswig-Holstein, Campus Kiel, Kiel, Germany; 2 Department of Biomedicine, University of Basel, Basel, Switzerland

**Keywords:** autoimmune blistering disease, biologic therapy, bullous pemphigoid, dupilumab, elderly patients, pruritus, refractory disease

## Abstract

Bullous pemphigoid (BP) is the most common autoimmune blistering disease affecting the skin. It is characterized by a type 2 immune-mediated pathogenesis. BP predominantly affects elderly patients who have multiple comorbidities, and treatment can be particularly challenging in refractory cases or when standard immunosuppressive therapies are limited due to toxicity or contraindications. Here, we present a case series of five patients with confirmed BP who were treated with off-label dupilumab. All five patients achieved sustained clinical improvement with dupilumab treatment, including reductions in disease activity and pruritus. No severe adverse events were reported, except for one case of manageable conjunctivitis. Dupilumab was well tolerated and resulted in marked clinical benefit in all five BP patients included in this case series. These findings support the growing evidence for dupilumab’s potential role as a safe and effective treatment option for BP. Further long-term clinical trials are needed to assess its disease-modifying potential.

## 1 Introduction

Bullous pemphigoid (BP) is the most prevalent autoimmune blistering disease affecting the skin (1, 2). It is characterized by a type 2 immune-mediated pathogenesis (3). Here, we present a case series of five patients with BP who were either unresponsive to standard therapy, experienced unacceptable adverse effects, or could not receive conventional immunosuppressive treatment due to toxicity or contraindications. All patients were subsequently treated with off-label dupilumab, a monoclonal anti-IL-4-receptor-alpha chain-specific IgG4 antibody blocking the signaling of IL-4 and IL-13 (Table 1).

**Table 1:** Patient profiles and outcomes following treatment with dupilumab.

Pat. no.	Sex (age)	Pre-existing conditions	Previous systemic therapies	Outcome under dupilumab treatment
1	Male (80s)	Melanoma stage IV Arterial hypertension Atrial fibrillation Chronic venous insufficiency BPH Hypothyroidism	GCS Dapsone Doxycycline	Rapid improvement IGA 2 to IGA 0 after 5 months
2	Male (80s)	History of bladder carcinoma Arterial hypertension BPH	GCS AZA Doxycycline	Rapid improvement IGA 3 to IGA 0 after 4 months
3	Female (90s)	Arterial hypertension Chronic kidney disease Chronic anemia Intraspinal empyema	GCS Dapsone AZA Doxycycline	Rapid improvement, rare appearance of blisters IGA 2 to IGA 1 after 2 months
4	Male (70s)	HIV Arterial hypertension Coronary heart disease Chronic kidney disease DM type II Polyneuropathy BPH	Dapsone	Rapid improvement IGA 2 to IGA 0 after 6 months
5	Male (70s)	Gynecomastia	GCS Dapsone AZA Doxycycline	Rapid improvement IGA 2 to IGA 0 after 13 months 3 months after dupilumab termination still IGA 0

BPH = benign prostatic hyperplasia, GCS = glucocorticosteroids, AZA = azathioprine, IGA0 = no symptoms, IGA1 = pruritus, no blistering, IGA2 = few blistering, IGA3 = moderate blistering, IGA4 = severe blistering (75% body surface), IGA5 = maximal affected skin.

## 2 Description of cases

Patient 1 was diagnosed with BP after receiving 12 cycles of pembrolizumab for stage IV malignant melanoma (AJCC classification; diagnosis in 2017; BP onset in 2022). With stable malignant disease, pembrolizumab was paused, and systemic treatment for BP was initiated with dapsone at a dosage of 100 mg daily in addition to topical clobetasol. Due to further progression of BP, oral prednisolone was introduced (initially 40 mg, which was tapered to 5 mg daily) in combination with doxycycline at a dosage of 200 mg daily. After an intermediate clinical response, new blisters appeared. Given the contraindication to immunosuppressants due to the patient’s history of melanoma, an off-label request for dupilumab treatment was submitted to the health insurance provider. In the interim, disease control was maintained with dexamethasone pulse therapy until insurance authorization for dupilumab was granted. Then, dexamethasone pulse therapy was stopped, and dupilumab was subsequently administered following the dosing regimen approved for atopic dermatitis. The patient’s skin condition remained stable, pruritus decreased, and no further blistering was observed within 5 months (IGA score improved from 2 to 0 after 5 months, Table 1).

Similarly, the skin conditions of patients 2 and 3 remained uncontrolled despite multiple therapeutic attempts, all of which had to be discontinued due to either insufficient efficacy or intolerable side effects. Following off-label treatment with dupilumab, both patients experienced sustained clinical improvement: Patient 2 improved from an IGA score of 3 to 0 within 4 months and patient 3 improved from an IGA score of 2 to 1 within 2 months of treatment (Table 1). In patients 4 and 5, due to insufficient response to conventional therapy with dapsone 100 mg or azathioprine 50 mg, an add-on treatment with dupilumab was initiated after receiving off-label authorization. The skin conditions of both patients improved significantly: Patient 4 improved from an IGA score of 2 to 0 after 6 months and patient 5 improved from an IGA score of 2 to 0 after 13 months, at which point azathioprine had been successfully discontinued without any reported exacerbations. All five patients tolerated dupilumab therapy well. Only patient 5 developed recurrent conjunctivitis during treatment with dupilumab. After discontinuing dupilumab, the patient’s ocular symptoms improved, and clinical BP remission was maintained for several months, accompanied by serological conversion to a negative autoantibody status. Table 2 summarizes prior therapies and reasons for changes in the therapy regimen for patients 2–5.

**Table 2:** Prior therapies and reasons for therapy regimen changes in patients 2–5.

Patient no.	Prior therapies before dupilumab (duration in months)	Reason for therapy regimen changes
2	Azathioprine (N/A)	Insufficient response
	Doxycycline (N/A)	Insufficient response
	Dexamethasone pulse (1)	No persistent improvement
	Doxycycline + prednisolone 15 mg – 5 mg (6)	Insufficient response
3	Prednisolone (1)	No persistent improvement
	Dexamethasone pulse (2)	No persistent improvement
	Dapsone (1)	Insufficient response
	Dexamethasone pulse (1)	No persistent improvement
	Dapsone + Azathioprine (1)	Insufficient response
	Dexamethasone pulse (2)	No persistent improvement
	Doxycycline (1)	Intolerance
4	Dapsone (1)	Insufficient response
5	Dapsone (N/A)	Insufficient response
	Doxycycline (N/A)	Insufficient response
	Prednisolone (N/A)	No persistent improvement
	Dapsone (11)	Secondary loss of efficacy
	Azathioprine (4)	Insufficient response

N/A = Not available.

## 3 Timeline

See Tables 1, 2.

## 4 Diagnostic assessment

All five patients met the classical diagnostic criteria for BP, including the clinical appearance of subepidermal blistering, linear deposition of autoantibodies along the basement membrane zone on direct immunofluorescence, and the presence of circulating autoantibodies against BP180 and/or BP230. In addition to cutaneous manifestations, patients 2 and 3 also presented with mucosal lesions, which similarly showed a good clinical response to dupilumab.

Dupilumab was administered following the atopic dermatitis regimen, with an initial dose of 600 mg subcutaneously, followed by 300 mg every 2 weeks.

## 5 Follow-up and outcomes

All five patients presented in this case series demonstrated sustained improvement in skin lesions and pruritus when treated with dupilumab. No re-hospitalizations were reported. The only notable adverse event was mild recurrent conjunctivitis in one patient, which resolved after discontinuation of dupilumab. Remission was maintained in that patient for several months without treatment.

Table 3 summarizes the concentrations of BP180 and BP230 antibodies at the time of BP diagnosis, before the initiation of dupilumab, and following the onset of treatment. In patients 2 and 5, a clear reduction in antibody concentrations was observed after commencing dupilumab. Patients 1 and 3 demonstrated an increase in antibody concentrations after treatment initiation despite showing good clinical responses. However, in patient 1, a gradual downward trend in antibody concentrations was observed over time. Patient 4 exhibited persistently negative results both at diagnosis and during dupilumab therapy.

**Table 3:** BP180 and BP230 antibody serum concentrations at the time of BP diagnosis, before the initiation of dupilumab, and after treatment with dupilumab.

Patient no.	Antibody concentration at diagnosis	Antibody concentration before the initiation of dupilumab	Antibody concentration after dupilumab treatment (no. of months after dupilumab treatment)
1	BP180: 14 U/mL* BP230: 0 U/ml*	BP180: 50 U/mL* BP230: 0 U/mL*	BP180: 432 U/mL* (4), 160 U/mL^#^ (7) BP230: 0 U/mL* (4), 0 U/mL* (7)
2	BP180: 218 U/mL* BP230: 35 U/mL*	BP180: N/A BP230: N/A	BP180: 35 U/ml^#^ (15) BP230: 14 U/mL^#^ (15)
3	BP180: 23 U/mL* BP230: 50 U/mL*	BP180: 46 U/mL* BP230: 38 U/mL*	BP180: 102 U/mL^#^ (8), 22 U/mL^#^ (17) BP230: <20 U/ml^#^ (8), <20 U/ml^#^ (17)
4	BP180: <9 U/ml* BP230: <9 U/ml* Split skin: positive	BP180: <9 U/mL* BP230: <9 U/mL*	BP180: <9 U/mL* (12) BP230: <9 U/mL* (12)
5	BP180: positive immunoblot BP230: negative immunoblot	BP180: positive immunoblot BP230: negative immunoblot	BP180: negative immunoblot (1) BP230: negative immunoblot (1)

* = Positive ≥ 9 U/mL. ^#^ = Positive ≥ 20 U/mL. N/A = Not available.

## 6 Discussion

This case series of real-world data from the University Hospital Schleswig-Holstein, Campus Kiel, shows both the efficacy and tolerability of dupilumab in five patients with difficult-to-treat BP. BP predominantly affects individuals over the age of 70, an age at which comorbidities commonly manifest and may limit the effectiveness and tolerability of conventional therapies, particularly when higher treatment doses are necessary. This is well recognized for systemic glucocorticoids, particularly with respect to hyperglycemia and/or hypertension, as also observed in our patient group. Furthermore, hematological, organ-specific (most commonly renal), or malignant pre-existing conditions may restrict the use of immunosuppressive agents such as azathioprine, mycophenolate mofetil, or rituximab. Particularly relevant in BP is the intolerance to dapsone, which is often insufficiently effective at low doses (<50 mg/day), while higher doses (>100 mg/day) may lead to reduced physical performance due to anemia and/or increased methemoglobin levels; this was observed in nearly all patients in our case series. In addition, gastrointestinal adverse effects associated with doxycycline, especially in the context of polypharmacy, may result in treatment discontinuation.

Our data support previously published case series and retrospective studies, primarily from the United States and Asia, suggesting the efficacy of dupilumab in the treatment of BP (4–6). We also investigated the evolution of BP180 and BP230 antibody concentrations, which have previously been shown to correlate well with disease severity (7), before and during dupilumab treatment. In some patients, we observed heterogeneous outcomes, which may be explained by differences in epitope specificity and IgG subclasses, as described in pemphigus previously (8). The exact mechanism by which dupilumab affects BP activity is not fully understood. However, recent research has demonstrated that dupilumab exerts effects on both immune cells and skin cells directly (9, 10). In the reported patients, the therapeutic response to dupilumab has remained sustained to date. None of the five patients were readmitted to the hospital. Whether dupilumab exerts a disease-modifying effect must be investigated in long-term, placebo-controlled clinical trials.

## Data Availability

The original contributions presented in the study are included in the article/supplementary material, further inquiries can be directed to the corresponding author.
